# Trends in Racial Disparities in Healthcare Expenditures Among Senior Medicare Fee-for-service Enrollees in 2007–2020

**DOI:** 10.1007/s40615-023-01832-x

**Published:** 2023-11-13

**Authors:** Seo H. Baik, Fitsum Baye, Clement J. McDonald

**Affiliations:** Lister Hill National Center for Biomedical Communications, National Library of Medicine, U.S. National Institutes of Health, 8600 Rockville Pike, Building 38A, Bethesda, MD 20894 USA

**Keywords:** Racial disparity, Trends, Healthcare expenditures, Medicare

## Abstract

Despite the universal healthcare coverages, racial disparities in healthcare expenditures among senior Medicare beneficiaries exist. A few studies explored how racial disparities in healthcare expenditures changed over past decades and how it affected differently across 4 minoritized races, by type of Medicare and poverty levels. We conducted a longitudinal study of 21 healthcare expenditures from senior Medicare fee-for-service enrollees to determine overall and secular trends in racial disparities in healthcare expenditures between 2007 and 2020, during which the Affordable Care Act (ACA) came into full force and the COVID-19 pandemic had begun. We found important disparities in healthcare expenditures across 4 minoritized races compared to Whites, even after adjusting for possible confounders for such disparities. Disparities between Hispanics/Asians and Whites were much greater than disparities between Blacks and Whites, in all Parts A, B, and D expenditures. This reality has not been sufficiently emphasized in the literature. Importantly, Black-White disparities in total Part B expenditure gradually worsened between 2007 and 2020, and Hispanic-White and Asian-White disparities worsened greatly during that time window. Health planners need to focus on these large disparities and develop methods to shrink them.

**Supplementary Information:**

The online version contains supplementary material available at 10.1007/s40615-023-01832-x.

## Introduction

Since its enactment in 1965, Medicare has provided a broad range of healthcare. Medicare hospital insurance program (Part A) covers services in a hospital, skilled nursing facility, hospice, and parts of care in home health. The Medical insurance program (Part B) covers doctors’ services, outpatient, and preventive care services. The prescription drug insurance program (Part D), newly implemented in 2006, covers prescription drugs and recommended vaccines [1]. In 2020, Medicare provides healthcare coverages to about 63 million people in the USA: 55 million seniors (age ≥ 65) and 8 million young adults with long-term disabilities [2]. The proportion of racial/ethnic minorities among Medicare beneficiaries continues to increase, growing from 22% in 2011 to 27% in 2021 [3]. Medicare has helped to mitigate disparities in healthcare by providing vulnerable populations with health insurance. However, many studies report that healthcare disparities in quality of care [4–6] and utilization [7–10] persist among Medicare populations. Disparities in healthcare are associated with limited/restricted access to appropriate medical care because of demographic (e.g., race and ethnicity, age, gender) [11–14], socio-economic (e.g., poverty, insurance type, education) [15–19], and geographical (place of residence) [15, 17, 20, 21] factors.

The Affordable Care Act (ACA) came into full force in 2014, and it had halved the rate of uninsured individuals by 2016, as a result of its expansion of Medicaid eligibility to young adults (age < 65) with income below 138% Federal Poverty Line (FPL) and its radical overhaul of the individual insurance market. Though the ACA’s primary targets were to improve health status among non-Medicare adults, the law also strengthened the Medicare program by fully covering annual preventive visits [22] and closing the “Donut Hole” coverage gap in Part D drug benefit [23].

Minoritized races experience higher rates of illness and death across a wide range of health conditions including heart disease, cancer, stroke, diabetes, flu and pneumonia, HIV/AIDS, and liver cirrhosis [24]. Compared to Whites, minoritized races were less likely to have a usual doctor visit, to receive mental health services, more likely to report poor health status and to die from diabetes and heart disease [25]. Hence, some healthcare expenditures to treat existing comorbidities would be higher for minoritized races. Healthcare expenditure is a function of the utilization of services, and lower healthcare expenditure among minoritized races could indicate lower utilization of healthcare services and thus could be a proxy for racial disparities in accessing healthcare. Unlike young adults, most elderly individuals have good healthcare coverage. Nonetheless, racial disparities in medical care expenditure [26–28] and prescription drug spending [29, 30] exist even among senior Medicare beneficiaries. Only a few studies focused on racial disparities in healthcare expenditure among seniors after the implementation of the ACA provisions [27], and these studies focused narrowly on medical expenditure as a whole. Aggregated medical expenditure, however, would not be sufficient to identify racial disparities in more granular categories of medical expenditures.

The US Centers for Medicare and Medicaid Services (CMS) Virtual Research Data Center (VRDC) [31] carries detailed cost information on medical encounters (1999–2020) and prescription drugs (2007–2020) from almost all (93%) non-institutionalized individuals age 65 or more [32]. As more complete healthcare cost data have been collected and the proportion of minoritized races has increased over decades, VRDC might have an ability to shed light on trends in racial disparities in various healthcare expenditures over the years. In this study, we examine Medicare expenditures by race from 2007 to 2020, during which ACA provisions and the COVID-19 pandemic occurred.

## Method

### Data Source and Study Population

We made use of 2007–2020 enrollment and medical and prescription drug claim records from a 20% random sample of Medicare beneficiaries. We limited eligible individuals to be those who first entitled to Medicare near age 65 (779–781 months old) and during the full availability of Part D benefit (i.e., 2007–2020). For each year between 2007 and 2020, we included all beneficiaries who ever enrolled in traditional Medicare fee-for-service (FFS) plans in analyses of medical expenditures. However, we excluded those solely enrolled in Medicare Advantage (MA or Part C) because the CMS does not have payment information for services provided to such patients. In the analysis of prescription drug expenditure, we only included those who ever enrolled in Part D plans. Furthermore, in each analysis of different service expenditures, we included only those who had any relevant Medicare expenditure in a given year. Therefore, the size of the resulting cohorts varied over the years (Appendix Table 1) and different service expenditures.

### Primary and Secondary Outcomes

The Medicare Master Beneficiary Summary File (MBSF) Cost and Use segment (https://resdac.org/cms-data/files/mbsf-cost-and-use) summarizes annual payments for 18 services covered by Part A (5 services: acute inpatient hospital, other inpatient hospital, skilled nursing facility, hospice, home health), Part B (12 services: hospital outpatient, ambulatory surgery center, anesthesia, Part B (drug, evaluation and management, physician, dialysis, other procedures, imaging, tests, durable medical equipment, other carrier), and Part D (prescription drugs) in a given calendar year. We first calculated the total expenditures of each of the 18 services. With some exceptions, a total expenditure is a sum of 3 parts, Medicare payment, beneficiary payment, and primary payer amount. We added per diem payment for total acute and other inpatient hospital expenditures and excluded beneficiary payments for total hospice and home health expenditures because Medicare beneficiaries pay nothing for hospice and home health services (Appendix Table 2). Total Part D drug expenditure was precalculated by the CMS. We report 3 grand total expenditures for Part A, Part B, and FFS as sums of 5, 12, and all 17 total expenditures, respectively. We included total FFS, Parts A, B, and D expenditures as our primary outcomes but also tracked expenditures for each of the 17 services as secondary outcomes. To account for the different enrollment durations in a given year, we annualized each of the 21 total expenditures but did not adjust them for inflation because we reported racial disparities as a ratio rather than a dollar amount.

### Covariates

The MBSF Base segment (https://resdac.org/cms-data/files/mbsf-base) includes patient’s demographics, area of residence, enrollment, and dual eligibility information. We used an enhanced race and ethnicity code developed by the Research Triangle Institute [33] to explore racial disparities in healthcare expenditures. Along with the self-reported race and ethnicity, this enhanced code implemented an algorithm to predict race and ethnicity based upon a list of common Hispanic and Asian/Pacific Islander surname and first names, and it was found to improve the accuracy of coding for non-black minorities [33]. In our analysis, we included 5 races, White, Black, Hispanic, Asian/Pacific Islander, and others, and compared each minoritized race to White (reference race). To account for secular trend, geography, and underlying differences in patient characteristics, our analyses were adjusted for fiscal year (2007–2020), area of residence (e.g., reside in a rural area), patient’s demographics (e.g., age and gender), socioeconomics, and overall health conditions. We used Medicare-Medicaid dual eligibility as a proxy for patient’s socioeconomic status. We utilized a monthly Medicare-Medicaid dual eligibility code to identify relevant individuals each month and separated patients into 3 groups: full, partial, and never dual groups in a given year. Dual eligible individuals have either full Medicaid coverage or Medicaid coverage of Medicare Parts A/B premium depending on their poverty level. In this study, the dual group included qualified Medicare beneficiaries (QMBs) with income < 100% FPL, specified low-income Medicare beneficiaries (SLMBs) with income between 100 and 120% FPL, and qualifying individuals (QIs) with income between 120 and 135% FPL [34]. To account for patients’ overall health conditions, we used the Charlson comorbidity index (CCI) [35] rather than individual chronic condition indicators to reduce the computational burden. The CCI is a sum of 23 different chronic conditions weighted by a scale of 1–6. It has been used to predict healthcare utilization cost to identify high-risk individuals to reduce high healthcare costs [36].

### Statistical Analysis

We took advantage of a generalized linear mixed regression model with gamma distribution and log link for 21 expenditure categories repeatedly measured for 14 years. We included a random intercept to account for correlations among healthcare expenditures within the same individuals and assessed the validity of our analytic model by using a residual analysis. We ran longitudinal regression analyses controlling not only for all covariates described above to mitigate confounding bias but also included interactions between calendar year, race, and dual eligibility group to explore racial inequalities by year and dual eligibility status. The exponentiated coefficient of the regression is the ratio of two means, e.g., indicating a multiplicative increase/decrease of a healthcare expenditure for a minoritized race from Whites. The ratio of 1 indicates no significant difference in a healthcare expenditure between two racial groups. We reported comparisons of each minoritized race against Whites, overall, by year, and by dual eligibility status.

## Results

### Patient Characteristics and Unadjusted Trends in Healthcare Expenditures

Table 1 presents a comparison of patient characteristics by race in the first (2007) and last (2020) years of our study. During our study period, the proportion of FFS enrollees dropped by 20%, from 66% in 2007 to 53% in 2020. In contrast, the proportion of Part D enrollees significantly increased from 38 to 74%. Females took slightly more than half of our study population. Minoritized races, barely 20% in 2007, reached 25% of the study population in 2020. The proportion of dual eligibles was higher among minoritized races than Whites (7.5–26.9% vs. 5.8% in 2020). Blacks were sicker; 9% of them had CCI score ≥ 3 in 2020. Other minoritized races were slightly healthier (1–3% lower in CCI scores ≥ 3) than Whites.

**Table 1:** Comparisons of baseline characteristics by race/ethnicity in 2007 and 2020

	2007				
	White	Black	Hispanic	Asian	Other
*N*	343,155	32,594	30,011	11,818	6514
FFS enrollees	229,570 (66.9)	22,525 (69.1)	18,524 (61.7)	6674 (56.5)	4074 (62.5)
Part D enrollees	127,535 (37.2)	12,018 (36.9)	13,633 (45.4)	5283 (44.7)	2256 (34.6)
Female	181,729 (53.0)	18,272 (56.1)	16,399 (54.6)	6313 (53.4)	3195 (49.0)
Rural	84,858 (24.7)	3875 (11.9)	2341 (7.8)	492 (4.2)	1560 (23.9)
Full dual	9349 (2.7)	3779 (11.6)	4151 (13.8)	1744 (14.8)	473 (7.3)
Partial dual	3063 (0.9)	1160 (3.6)	1547 (5.2)	602 (5.1)	144 (2.2)
Never dual	330,743 (96.4)	27,655 (84.8)	24,313 (81.0)	9472 (80.1)	5897 (90.5)
Age 65–69	343,155 (100.0)	32,594 (100.0)	30,011 (100.0)	11,818 (100.0)	6514 (100.0)
Age 70–74	0 (0.0)	0 (0.0)	0 (0.0)	0 (0.0)	0 (0.0)
Age 75 +	0 (0.0)	0 (0.0)	0 (0.0)	0 (0.0)	0 (0.0)
CCI = 0	302,662 (88.2)	27,021 (82.9)	26,431 (88.1)	10,507 (88.9)	5689 (87.3)
CCI = 1	22,983 (6.7)	2936 (9.0)	2130 (7.1)	805 (6.8)	490 (7.5)
CCI = 2	10,939 (3.2)	1402 (4.3)	828 (2.8)	310 (2.6)	180 (2.8)
CCI = 3 +	6571 (1.9)	1235 (3.8)	622 (2.1)	196 (1.7)	155 (2.4)
	2020				
	White	Black	Hispanic	Asian	Other
*N*	5,060,234	552,696	562,455	257,267	264,634
FFS enrollees	2,845,693 (56.2)	234,900 (42.5)	189,521 (33.7)	105,014 (40.8)	148,396 (56.1)
Part D enrollees	3,737,770 (73.9)	400,339 (72.4)	434,207 (77.2)	194,880 (75.8)	188,171 (71.1)
Female	2,749,394 (54.3)	318,130 (57.6)	312,412 (55.5)	145,466 (56.5)	108,980 (41.2)
Rural	1,225,710 (24.2)	65,480 (11.8)	45,583 (8.1)	9379 (3.6)	51,333 (19.4)
Full dual	259,073 (5.1)	109,180 (19.8)	137,454 (24.4)	59,606 (23.2)	17,604 (6.7)
Partial dual	35,884 (0.7)	12,452 (2.3)	14,082 (2.5)	4455 (1.7)	2034 (0.8)
Never Dual	4,765,277 (94.2)	431,064 (78.0)	410,919 (73.1)	193,206 (75.1)	244,996 (92.6)
Age 65–69	2,095,635 (41.4)	255,531 (46.2)	257,521 (45.8)	122,010 (47.4)	117,460 (44.4)
Age 70–74	1,843,248 (36.4)	194,301 (35.2)	195,911 (34.8)	90,335 (35.1)	120,263 (45.4)
Age 75 +	1,121,351 (22.2)	102,864 (18.6)	109,023 (19.4)	44,922 (17.5)	26,911 (10.2)
CCI = 0	3,841,549 (75.9)	423,183 (76.6)	475,443 (84.5)	210,135 (81.7)	203,769 (77.0)
CCI = 1	497,033 (9.8)	46,894 (8.5)	37,188 (6.6)	22,326 (8.7)	25,681 (9.7)
CCI = 2	343,287 (6.8)	32,972 (6.0)	21,901 (3.9)	11,686 (4.5)	17,577 (6.6)
CCI = 3 +	378,365 (7.5)	49,647 (9.0)	27,923 (5.0)	13,120 (5.1)	17,607 (6.7)

Data are presented as no. (%) of patients unless otherwise noted

*FFS*, fee-for-service; *CCI*, Charlson comorbidity index

**Fig. 1:**
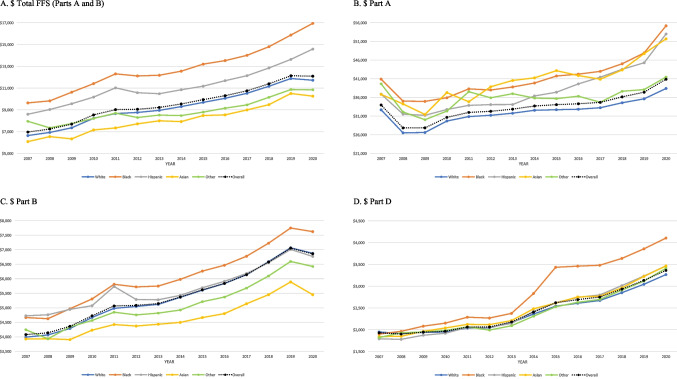
Unadjusted trends in 4 Medicare healthcare expenditures by race and ethnicity

Overall, total FFS expenditure per person per year increased from $6961 in 2007 to $12,093 in 2020. Without adjusting for confounders, Blacks had 30–40% greater total FFS expenditure ($9639 in 2007 and $16,943 in 2020) than the population average (Fig. 1A). This unadjusted difference stood out more in total Part A expenditure (17–25%) than total Part B expenditure (10–15%) (Fig. 1B and C). For Part D prescription drug expenditure, all races were numerically close until 2013. However, Part D expenditure for Blacks ballooned starting in 2014, the first year of the ACA Medicaid expansion, and became greater than other races thereafter (Fig. 1D). Interestingly, total FFS expenditure for Blacks and Hispanics increased further in 2020 (the first year of the COVID-19 pandemic), possibly because those two populations were more likely to be infected (Fig. 1A).

### Primary Analyses

First off, residual analyses show that residuals are normally distributed with mean zero and equal variance, and thus, our analytic model fits the data well (Appendix Fig. 1). In Table 2, we present multiplicative changes in 4 total expenditures associated with each covariate in the regression analysis. We report the change in terms of a ratio of two adjusted means of a total expenditure and interpret it as the percent increase/decrease from the reference group. Being female and older (70 +) were associated with a significantly higher total FFS expenditure than their counterparts, by + 16.7% and + 8.7–17.7%, respectively. The same pattern was observed in the total Parts B and D expenditures and the opposite pattern for the total Part A expenditure. Living in rural area was associated with significantly lower healthcare expenditures. CCI score was the strongest predictor of healthcare expenditures among all covariates. Compared to those with CCI score = 0, all healthcare expenditures exponentially increased with CCI score ≥ 1: total FFS expenditure for CCI score = 1, 2, 3 + were + 105%, + 224%, + 761% more than that for CCI score = 0, respectively (Table 2).

**Table 2:** Multiplicative changes of 4 primary outcomes associated covariates

	$ total FFS cost	$ Part A	$ Part B	$ Part D drug
Female vs. male	1.167 (1.165, 1.169)	0.936 (0.933, 0.938)	1.185 (1.183, 1.187)	1.054 (1.051, 1.057)
70–74 vs. 65–69	1.087 (1.086, 1.089)	0.931 (0.929, 0.934)	1.075 (1.074, 1.077)	1.112 (1.110, 1.113)
75 + vs. 65–69	1.177 (1.174, 1.180)	0.952 (0.947, 0.956)	1.130 (1.127, 1.132)	1.182 (1.179, 1.184)
Rural vs. not	0.913 (0.911, 0.915)	0.936 (0.934, 0.939)	0.920 (0.918, 0.921)	0.940 (0.938, 0.942)
CCI = 1 vs. 0	2.054 (2.051, 2.058)	1.053 (1.049, 1.057)	1.826 (1.824, 1.829)	1.372 (1.370, 1.374)
CCI = 2 vs. 0	3.237 (3.231, 3.243)	1.190 (1.186, 1.195)	2.650 (2.646, 2.654)	1.472 (1.469, 1.475)
CCI = 3 + vs. 0	8.612 (8.595, 8.628)	1.951 (1.944, 1.957)	5.264 (5.254, 5.273)	1.984 (1.980, 1.988)
Full dual vs. never	1.433 (1.425, 1.442)	1.081 (1.070, 1.093)	1.342 (1.335, 1.349)	1.578 (1.569, 1.587)
Partial dual vs. never	1.443 (1.431, 1.456)	1.389 (1.366, 1.413)	1.259 (1.250, 1.269)	1.227 (1.217, 1.236)

Data are presented as a ratio of estimated annualized spendings (95% confidence interval)

*FFS*, fee-for-service; *CCI*, Charlson comorbidity index

### Trends in Racial Disparities

**Fig. 2:**
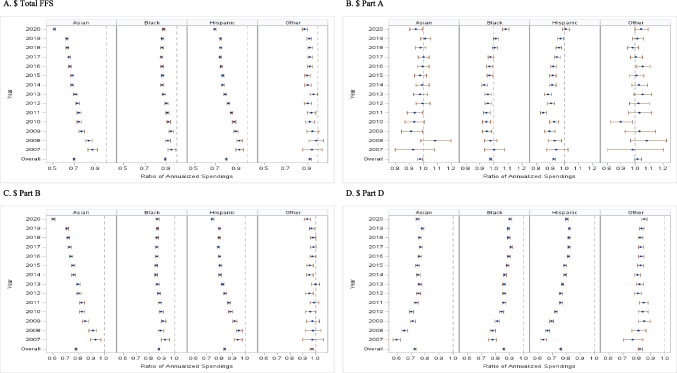
Trends in racial disparities in 4 healthcare expenditures

In Table 3 and Fig. 2, we present comparisons of total healthcare expenditures between each minoritized race and Whites, across years and by year, adjusting for all covariates. All minoritized races, overall, had significantly lower healthcare expenditures than Whites after controlling for different patient characteristics. Contrary to unadjusted comparisons, Blacks had − 11% lower total FFS expenditure than Whites. This imbalance was disproportionately wider in Part B (− 12%) than in Part A (− 2%) expenditures. Moreover, there were important variations in expenditures for each of the 12 Part B services. Blacks had lower expenditures than Whites in 7 out of 12 Part B services: ambulatory surgery (− 7%), Part B drugs (− 11%), anesthesia (− 4%), other procedures (− 17%), imaging (− 10%), tests (− 8%), and Part B physician (− 9%). They had higher expenditures in hospital outpatients (+ 1%), evaluation and management (+ 3.5%), dialysis (+ 55%), durable medical equipment (+ 5%), and other Part B carrier services (+ 14%) (Table 3). Between 2007 and 2020, the disparities among Blacks in Part A persisted at around − 5%, worsened moderately in Part B from − 7 to − 13%, and improved in Part D from − 22 to − 9% (Fig. 2B, C, and D).

**Table 3:** Overall disparities in 21 healthcare expenditures, comparing 4 minoritized races to Whites

	Black	Hispanic	Asian	Others
Total FFS cost	0.890 (0.884, 0.896)	0.805 (0.800, 0.810)	0.699 (0.693, 0.706)	0.927 (0.914, 0.941)
Part A total	0.977 (0.969, 0.986)	0.927 (0.917, 0.936)	0.977 (0.958, 0.995)	1.019 (0.995, 1.043)
Part B total	0.878 (0.873, 0.883)	0.840 (0.836, 0.845)	0.781 (0.775, 0.788)	0.971 (0.959, 0.984)
Part D drug	0.859 (0.853, 0.865)	0.762 (0.757, 0.767)	0.729 (0.722, 0.736)	0.826 (0.814, 0.839)
Acute inpatient	1.044 (1.035, 1.052)	1.093 (1.082, 1.103)	1.158 (1.138, 1.179)	1.079 (1.056, 1.102)
Other inpatient costs	1.163 (1.138, 1.189)	1.157 (1.123, 1.192)	1.230 (1.152, 1.314)	1.053 (0.989, 1.122)
Skilled nursing facility	1.178 (1.154, 1.203)	1.090 (1.057, 1.124)	1.103 (1.042, 1.167)	1.033 (0.970, 1.100)
Hospice	1.099 (1.055, 1.144)	1.075 (1.020, 1.133)	1.078 (0.963, 1.207)	0.971 (0.866, 1.090)
Home health	1.153 (1.139, 1.167)	1.233 (1.217, 1.250)	1.117 (1.087, 1.147)	1.070 (1.018, 1.124)
Hospital outpatient	1.010 (1.002, 1.018)	1.028 (1.019, 1.036)	0.899 (0.887, 0.911)	1.082 (1.062, 1.103)
Ambulatory surgery	0.928 (0.914, 0.942)	1.051 (1.038, 1.065)	1.040 (1.021, 1.058)	1.074 (1.040, 1.110)
Part B drug	0.889 (0.880, 0.899)	0.933 (0.924, 0.943)	0.976 (0.963, 0.989)	0.990 (0.966, 1.015)
Evaluation and management	1.035 (1.029, 1.041)	0.939 (0.933, 0.945)	0.861 (0.854, 0.869)	0.907 (0.895, 0.920)
Anesthesia	0.955 (0.949, 0.962)	1.004 (0.997, 1.011)	0.954 (0.943, 0.964)	1.013 (0.996, 1.031)
Dialysis	1.548 (1.472, 1.629)	1.605 (1.508, 1.707)	1.551 (1.407, 1.709)	N/A
Other procedures	0.832 (0.825, 0.840)	0.986 (0.977, 0.995)	0.995 (0.982, 1.009)	1.059 (1.036, 1.084)
Imaging	0.903 (0.898, 0.909)	1.082 (1.075, 1.089)	1.142 (1.131, 1.152)	0.990 (0.974, 1.005)
Tests	0.916 (0.910, 0.922)	1.086 (1.079, 1.093)	1.112 (1.101, 1.123)	0.988 (0.972, 1.005)
Durable medical equipment	1.050 (1.038, 1.063)	0.841 (0.831, 0.852)	0.643 (0.630, 0.655)	0.909 (0.882, 0.937)
Other Part B carrier	1.135 (1.119, 1.152)	0.853 (0.838, 0.868)	0.792 (0.770, 0.814)	1.103 (1.060, 1.147)
Part B physician	0.912 (0.908, 0.916)	0.999 (0.994, 1.003)	1.106 (1.099, 1.113)	1.002 (0.993, 1.012)

Data are presented as a ratio of estimated annualized spendings (95% confidence interval)

*FFS*, fee-for-service; *N/A*, not applicable, excluded from the analysis due to small counts

Compared to Black-White disparities, Hispanic-White and Asian-White disparities were much greater in general and worsened over years. Overall, Hispanics and Asians had − 19% and − 30% lower total FFS expenditures than Whites, respectively. Hispanic-White and Asian-White disparities in total Part B (− 16% and − 22%) and Part D (− 24% and − 27%), expenditures were especially pronounced. Among Part B services, both Hispanics and Asians had higher expenditures than Whites only in ambulatory surgery (by + 4–5%), dialysis (by + 55–61%), imaging (by + 8–14%), and tests (by + 9–11%) (Table 3). Between 2007 and 2020, while differences in Part D expenditure shrunk, from − 36 to − 20% for Hispanic-Whites and from − 40 to − 24% for Asian-White, disparities in total Part B expenditure grew from − 5 to − 25% and from − 6 to − 40%, respectively (Fig. 2C and D). Interestingly, compared to Black-White disparities, disparities in total Part B expenditures grew significantly wider in 2020 (the first year of the COVID-19 pandemic), from − 20% in 2019 to − 25% in 2020 for Hispanic-White and − 29% in 2019 to − 40% in 2020 for Asian-White, especially in expenditures for hospital outpatients, evaluation and management, and tests (Appendix Fig. 2).

### Trends in Racial Disparities by Dual Eligibility

Table 4 presents the same sets of results above separated by the dual eligibility. Overall, being dual was significantly associated with more FFS expenditure than non-dual (Table 2)—with + 43% more for full dual and + 44% more for partially dual enrollees. Though racial disparities in healthcare expenditures continued across different dual-eligibility statuses, the differences diminished among dual-eligible individuals. On average, fully dual-eligible Blacks had − 4% less total FFS expenditure than fully dual-eligible Whites; the same metric increased further to − 11% for partially dual Blacks and − 17% for never dual Blacks (Table 4). Between 2007 and 2020, disparities in total FFS expenditure among full dual Blacks persisted at around − 5% but disparities among non-dual Blacks worsened from − 9 to − 13% (Appendix Fig. 3).

**Table 4:** Overall disparities in 4 healthcare expenditures, comparing 4 minoritized races to Whites, across different dual eligibility

		Black	Hispanic	Asian	Others
$ total FFS	Full dual	0.955 (0.946, 0.964)	0.876 (0.868, 0.884)	0.719 (0.710, 0.728)	0.974 (0.953, 0.996)
	Partial dual	0.885 (0.873, 0.898)	0.741 (0.732, 0.751)	0.643 (0.630, 0.656)	0.858 (0.829, 0.887)
	Non-dual	0.833 (0.830, 0.837)	0.803 (0.799, 0.807)	0.740 (0.734, 0.745)	0.954 (0.947, 0.961)
$ Part A	Full dual	1.022 (1.009, 1.035)	0.912 (0.900, 0.924)	0.855 (0.832, 0.878)	1.011 (0.977, 1.046)
	Partial dual	0.948 (0.927, 0.969)	0.880 (0.859, 0.902)	1.013 (0.968, 1.060)	0.975 (0.919, 1.034)
	Non-dual	0.964 (0.956, 0.971)	0.991 (0.981, 1.002)	1.076 (1.055, 1.097)	1.073 (1.055, 1.090)
$ Part B	Full dual	0.932 (0.924, 0.940)	0.918 (0.911, 0.926)	0.830 (0.820, 0.839)	1.025 (1.005, 1.045)
	Partial dual	0.880 (0.869, 0.891)	0.811 (0.802, 0.821)	0.756 (0.743, 0.770)	0.929 (0.902, 0.957)
	Non-dual	0.824 (0.821, 0.827)	0.797 (0.793, 0.801)	0.761 (0.755, 0.766)	0.963 (0.957, 0.969)
$ Part D drug	Full dual	0.840 (0.832, 0.848)	0.735 (0.728, 0.741)	0.759 (0.749, 0.768)	0.772 (0.755, 0.788)
	Partial dual	0.859 (0.848, 0.869)	0.759 (0.750, 0.768)	0.740 (0.728, 0.753)	0.819 (0.794, 0.845)
	Non-dual	0.879 (0.874, 0.884)	0.793 (0.788, 0.798)	0.690 (0.683, 0.696)	0.892 (0.884, 0.901)

Data are presented as a ratio of estimated annualized spendings (95% confidence interval)

*FFS*, fee-for-service

Across different dual eligibilities, Hispanic-White and Asian-White disparities were significantly larger than Black-White disparities, and such disparities occurred in total FFS, as well as in Parts B and D expenditures. Overall, full, partial, and no dual Hispanics had − 12%, − 26%, and − 20% lower total FFS expenditure than Whites, and differences between Asians and Whites were even wider, − 28%, − 36%, and − 26% for full, partial, and no dual, respectively. However, unlike Black-White disparity, the size of the disparity did not increase linearly with the dual eligibilities. During 2007 to 2020, full dual Hispanic-White disparities in total FFS expenditure gradually increased from an insignificant + 2% in 2007 to a significant − 25% in 2020. The imbalance for full dual Asian-White was even greater and that difference grew to a significant − 48% in 2020. Hispanic-White and Asian-White disparities in total FFS and Part B had worsened at all levels of dual eligibility (Appendix Fig. 3).

### Sensitivity Analysis

We conducted two sets of sensitivity analyses to explore how gender and the implementation of ACA affected racial disparities in healthcare expenditures. In a set of sensitivity analyses stratified by gender, Asian-White disparities in all Parts A, B, and D expenditures were numerically greater among female than male. Between Hispanics and Whites, disparities among female Hispanics were greater in Part A and lesser in Parts B and D expenditures than male Hispanics. Differences between female and male Blacks in disparities in Part A and B expenditures were very small, but that in Part D expenditure was greater among male than female Blacks (Appendix Table 3). In another set of sensitivity analyses testing racial disparities before and after the implementation of ACA (i.e., difference-in-difference), racial disparities in Part B expenditures after ACA (2014–2020), compared to before ACA (2007–2013), had significantly widened by +8.9%, +1.9%, and +6.1% for Asians, Blacks, and Hispanics respectively. In contrast, disparities in Part D expenditure had significantly shrunk by -4%, -3.7%, and -6.0% for Asians, Blacks, and Hispanics respectively (Appendix Table 4).

## Discussion

In this study, we used 14-year longitudinal records of 21 healthcare expenditures from senior Medicare beneficiaries to explore trends in racial disparities in healthcare expenditures between 2007 and 2020. Our findings were adjusted for calendar year, geography, patient’s demographics, socio-economic status, and patient’s health risk score to mitigate possible confounders of racial disparities in healthcare expenditures. Racial disparities in healthcare expenditures persisted importantly after the adjustment for such covariates. The magnitude of disparities varied across different races/ethnicities. Notably, differences between Hispanics/Asians and Whites were much greater than differences between Blacks and Whites in all FFS, Parts A, B, and D expenditures. This reality has not been emphasized sufficiently in the literature. While Black-White disparities in total FFS expenditure were consistently at around − 10% between 2007 and 2020, Hispanic-White and Asian-White disparities worsened greatly from − 7 to − 30% and − 13% and − 48%, respectively.

We also found that the racial disparities affect healthcare expenditures differently by type of Medicare (i.e., Parts A, B, and D) and levels of dual Medicare-Medicaid eligibility. Racial disparities were more prominent in Parts B and D than in Part A expenditure, and the size of such disparities diminished among those who were dually eligible. Our additional analyses further suggest that gender also differently affected racial disparities. Female Asians had greater gaps in all Parts A, B, and D expenditures than male Asians, only in Part A expenditure for female Hispanics, and none for female Blacks. Delaying care [37] and lack of healthcare access due to family structure, language, and cultural factors [38] among female non-black minorities might explain such gender and racial disparities in healthcare expenditures.

Importantly, racial disparities in Part B expenditure either persisted or widened in the last 14 years. In contrast, racial disparities in Part D expenditures diminished importantly during that time frame. Substantial decreases in the use of preventive visits not covered by Medicare (from 16.0 to 5.5%) [39] and significant decreases in out-of-pocket spending for prescription drugs [40] after the implementation of ACA might explain these opposite trends. Our sensitivity analyses further suggest that racial disparities in Part B expenditures had become wider for Asians and Hispanics than Blacks. During the first year of the COVID-19 pandemic (i.e., 2020) compared to the preceding year (i.e., 2019), disparities in Part B expenditure grew even wider for Hispanic/Asian-White than Black-White. Specifically, among 12 Part B services, Hispanic/Asian-White disparities in expenditures for preventive services (e.g., hospital outpatient, evaluation and management, imaging, and tests) stood out in 2020 possibly due to delayed or missed preventive care [41], declines in wellness visits, checkups, and cancer screenings [42], a rapid increase in health care delivery via telehealth [43], and challenges in accommodating safety equipment and medical staffing [44, 45].

Previous studies often focused on racial disparities between Blacks and Whites [27, 28]. Other minoritized races had often been excluded or lumped together because of inaccuracies in self-reported race and ethnicity and the relatively small size of other minoritized races in the analysis. We used an enhanced race code that significantly increases the sensitivity for both Hispanics and Asians [46] and compared each of the 4 minoritized races to Whites.

Previous studies, based on Medical Expenditure Panel Survey data, reported racial disparities in healthcare expenditures but failed to highlight disparities in either Hispanics or Asians [47–50]. One study with 5 minoritized races reported that the racial disparities vary among 6 different types of medical care expenditures [12] but did not report how racial disparities in healthcare expenditure changed over time. A recent longitudinal study, also based on survey data, only reported that Black-White disparities in total healthcare expenditure had persisted over decades and widened in recent years [27]. Our results, based on insurance claim data, confirm those survey-based findings and further suggest important variations among 4 minoritized races over 14 years.

During our study epoch, the ACA provisions came into full force in 2014 and racial disparities in coverage have shrunk [51, 52]. After the implementation of the ACA provisions, the rate of annual preventive visits (Part B) increased from 1.4 to 27.% [39] and out-of-pocket spending for prescription drug (Part D) decreased [23]. However, we found that it has selectively mitigated disparities in healthcare expenditures. Racial disparities in Parts B and D healthcare expenditures persisted in 2014 and forward. Moreover, disparities in Part B expenditure had continued or worsened, while disparities in Part D expenditure had shrunk.

Our study has some limitations. First, we did not include Medicare beneficiaries who were solely enrolled in MA plans. The CMS does not have payment information for services covered by MA plans. The proportion of MA enrollees increased from 37% in 2007 to 47% in 2020, which may affect the generalizability of our findings. Second, in order to control for an individual’s health risk, we included Charlson comorbidity index (CCI) [35] rather than individual chronic condition flags [53]. Though the healthcare expenditure would be closely related to individual chronic conditions rather than aggregated measure of health status, we used CCI instead to reduce the computational burden.

## Conclusions

Despite the universal healthcare coverages, racial disparities in healthcare expenditures among senior Medicare beneficiaries exist. Only a few studies explored how racial disparities in healthcare expenditures changed over past decades and how it affected differently by 4 minoritized races. Our findings indicate important racial disparities among elderly Medicare beneficiaries in 2007–2020. Disparities are substantially greater among Hispanics and Asians than Blacks and have gotten worse over time. Previous studies focused mostly on Black-White disparities in healthcare expenditures [27, 50] and overlooked larger disparities between Whites and Hispanics/Asians (who make up a greater proportion of the US population). Health planners need to focus on these large disparities and develop methods to shrink them.

## Supplementary Information

Below is the link to the electronic supplementary material.

Supplementary file1 (PPTX 396 KB)

Supplementary file2 (PPTX 366 KB)

Supplementary file3 (PPTX 288 KB)

Supplementary file4 (DOCX 23 KB)

## Data Availability

The minimal data set relevant to this study is included and all data can be used without restriction. As for raw data, CMS did not allow the authors to download or distribute any patient level data. The data stayed in their machine and the authors analyzed it with software they provide on their machine. The shared detailed statistical data should be sufficient for anyone to verify the study’s results. If researchers wish to access the raw data, they can contact the CMS Virtual Research Data Center. Data access requires the payment of a fee.
