# Supplementary material for: Trends in Racial Disparities in Healthcare Expenditures Among Senior Medicare Fee-for-service Enrollees in 2007–2020
Source: J Racial Ethn Health Disparities. 2023 Nov 13;11(6):3807–17. doi: 10.1007/s40615-023-01832-x (PMC11564202; doi:10.1007/s40615-023-01832-x)
Supplement: Supplementary file 1 — Supplementary file1 (PPTX 396 KB) [file 40615_2023_1832_MOESM1_ESM.pptx]

## Slide 1
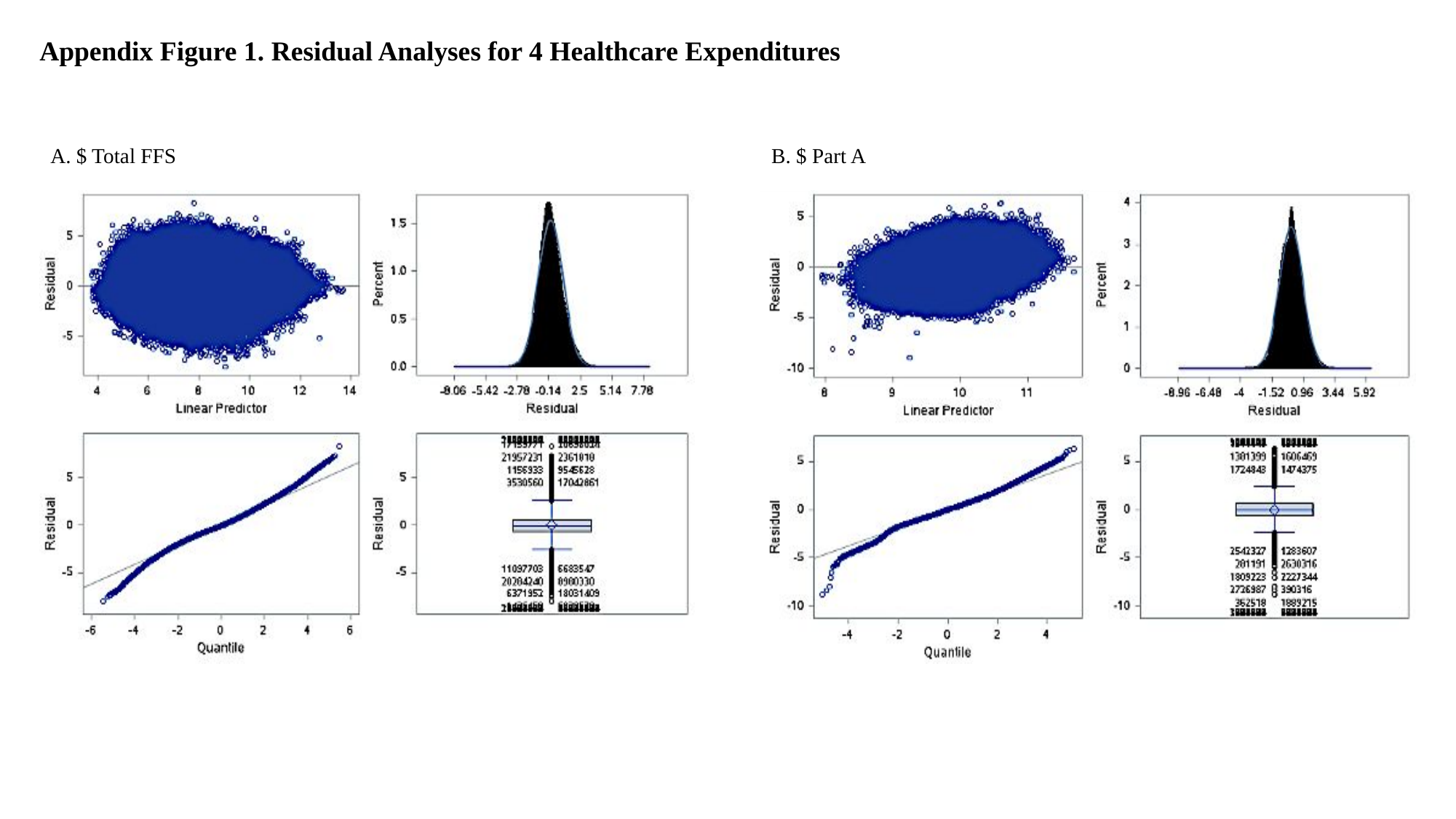

Appendix Figure 1. Residual Analyses for 4 Healthcare Expenditures
A. $ Total FFS
B. $ Part A

## Slide 2
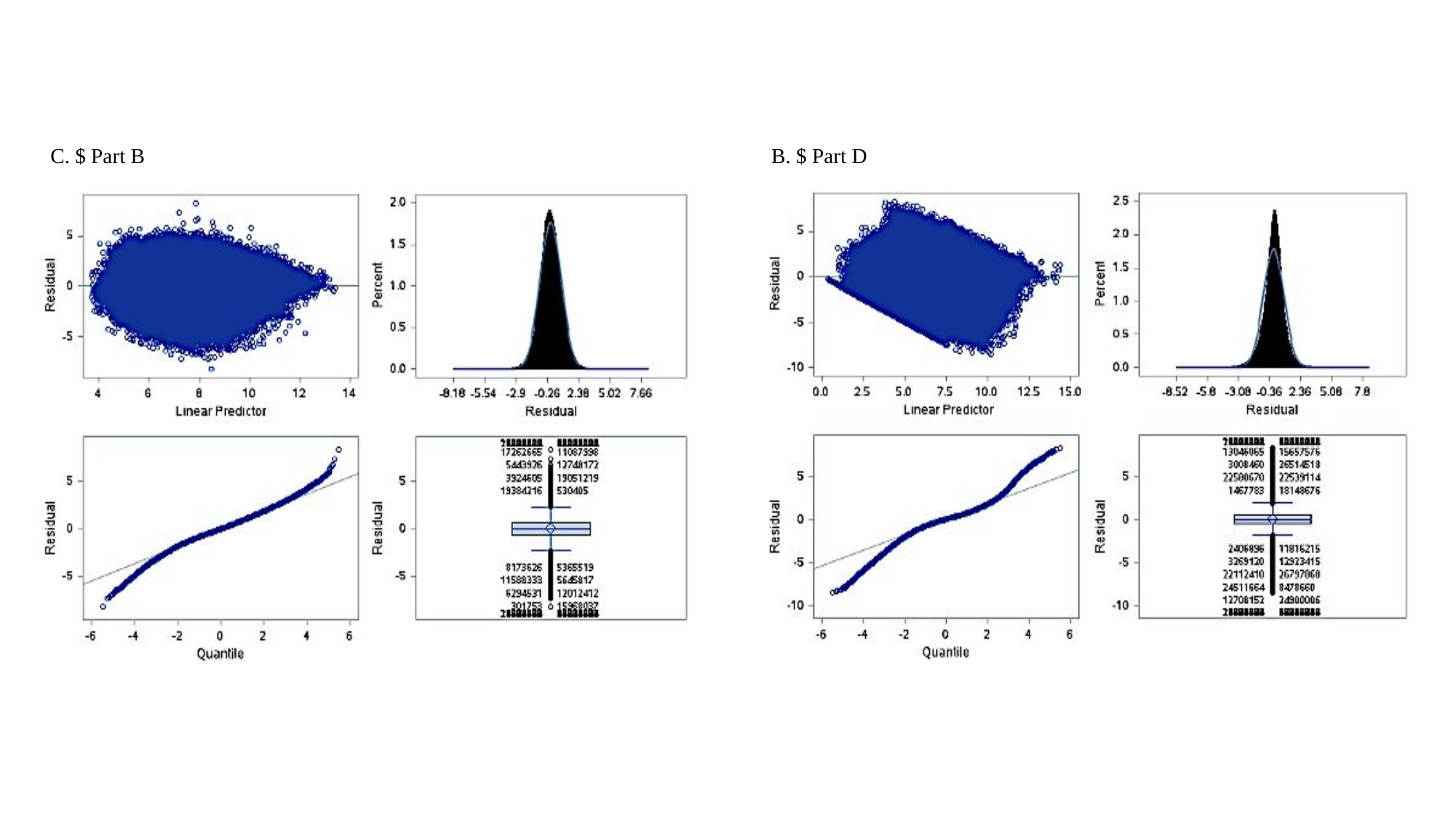

C. $ Part B
B. $ Part D
