# Supplementary material for: Trends in Racial Disparities in Healthcare Expenditures Among Senior Medicare Fee-for-service Enrollees in 2007–2020
Source: J Racial Ethn Health Disparities. 2023 Nov 13;11(6):3807–17. doi: 10.1007/s40615-023-01832-x (PMC11564202; doi:10.1007/s40615-023-01832-x)
Supplement: Supplementary file 2 — Supplementary file2 (PPTX 366 KB) [file 40615_2023_1832_MOESM2_ESM.pptx]

## Slide 1
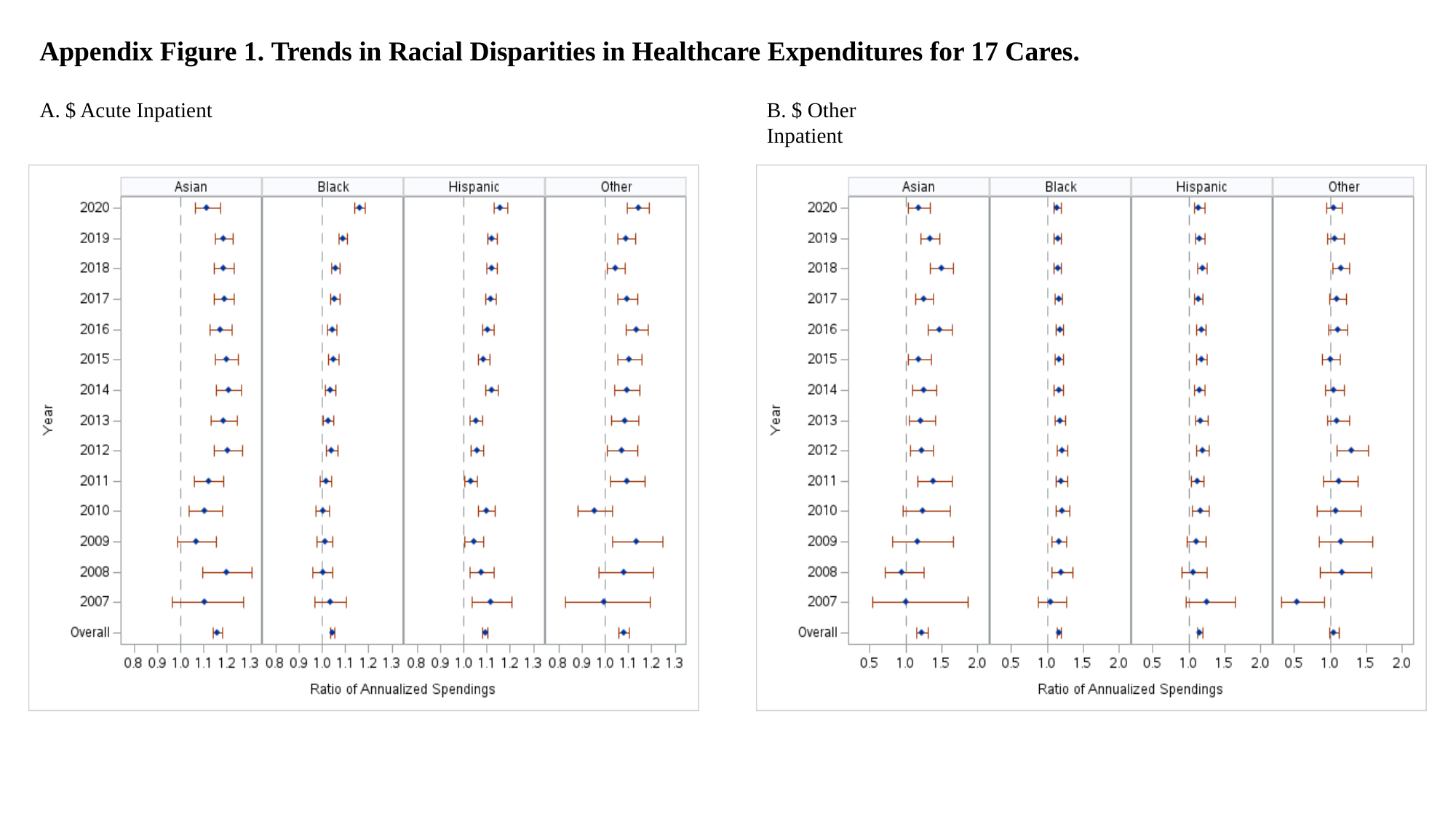

Appendix Figure 1. Trends in Racial Disparities in Healthcare Expenditures for 17 Cares.
B. $ Other Inpatient
A. $ Acute Inpatient

## Slide 2
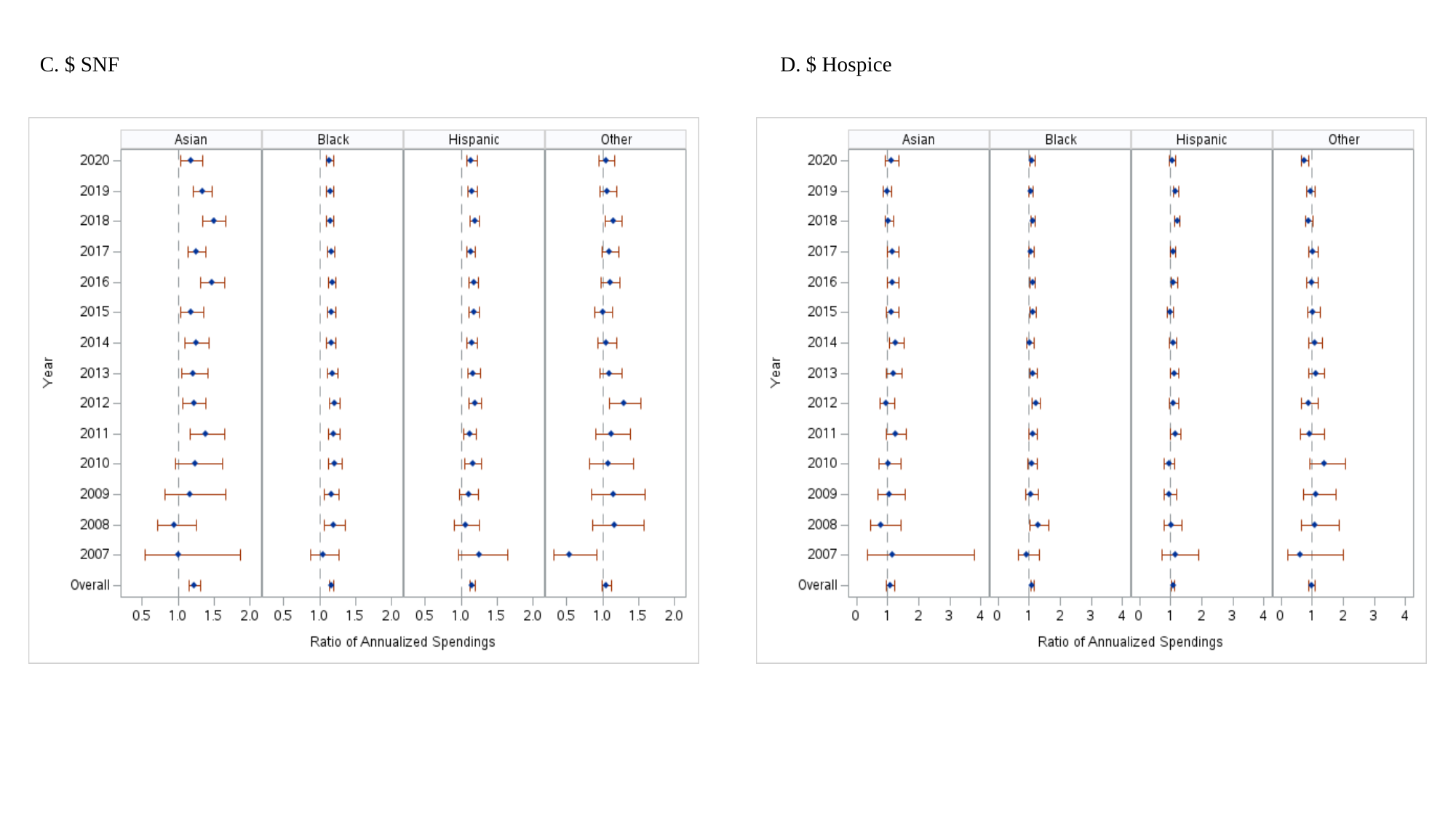

D. $ Hospice
C. $ SNF

## Slide 3
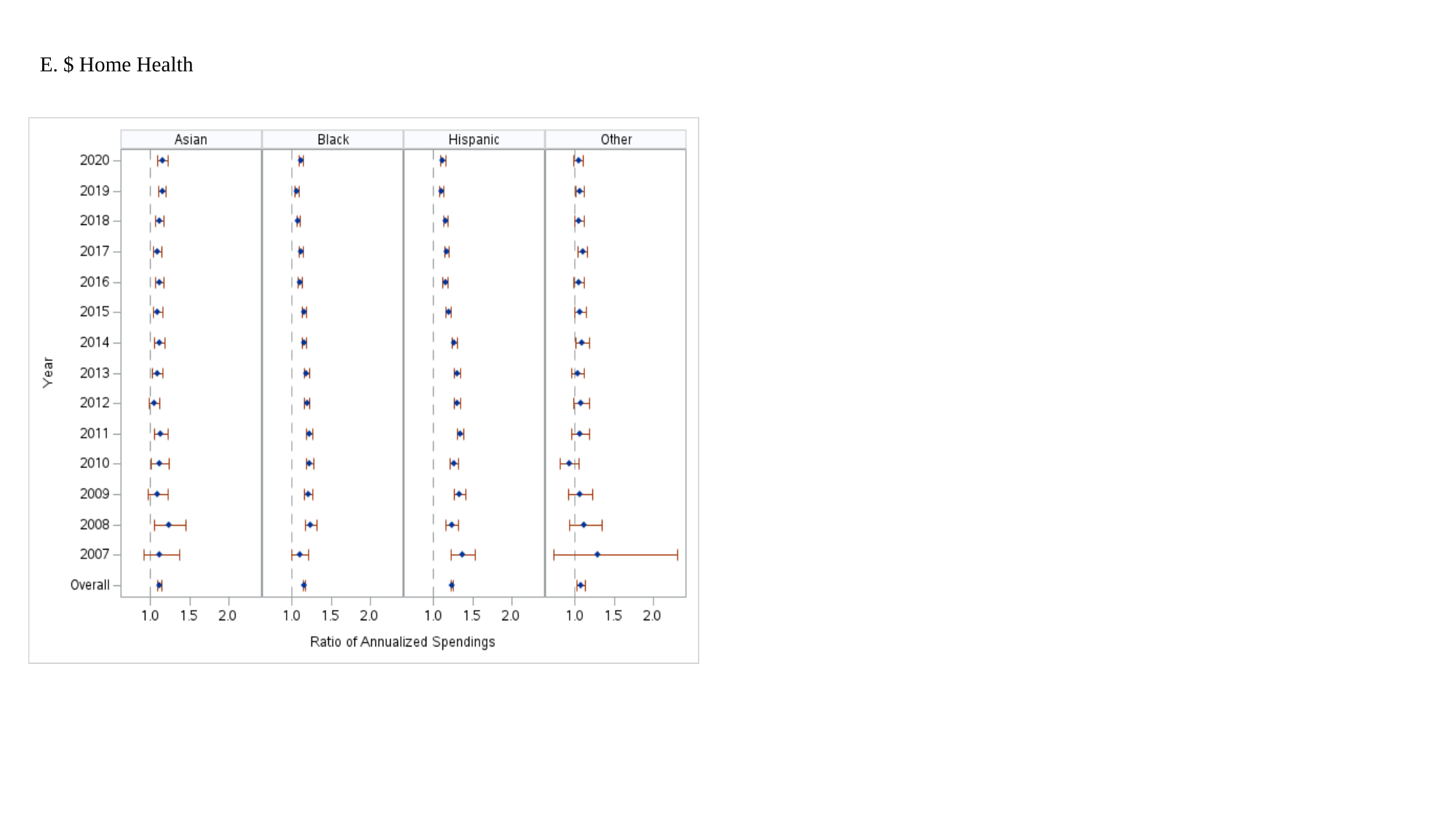

E. $ Home Health

## Slide 4
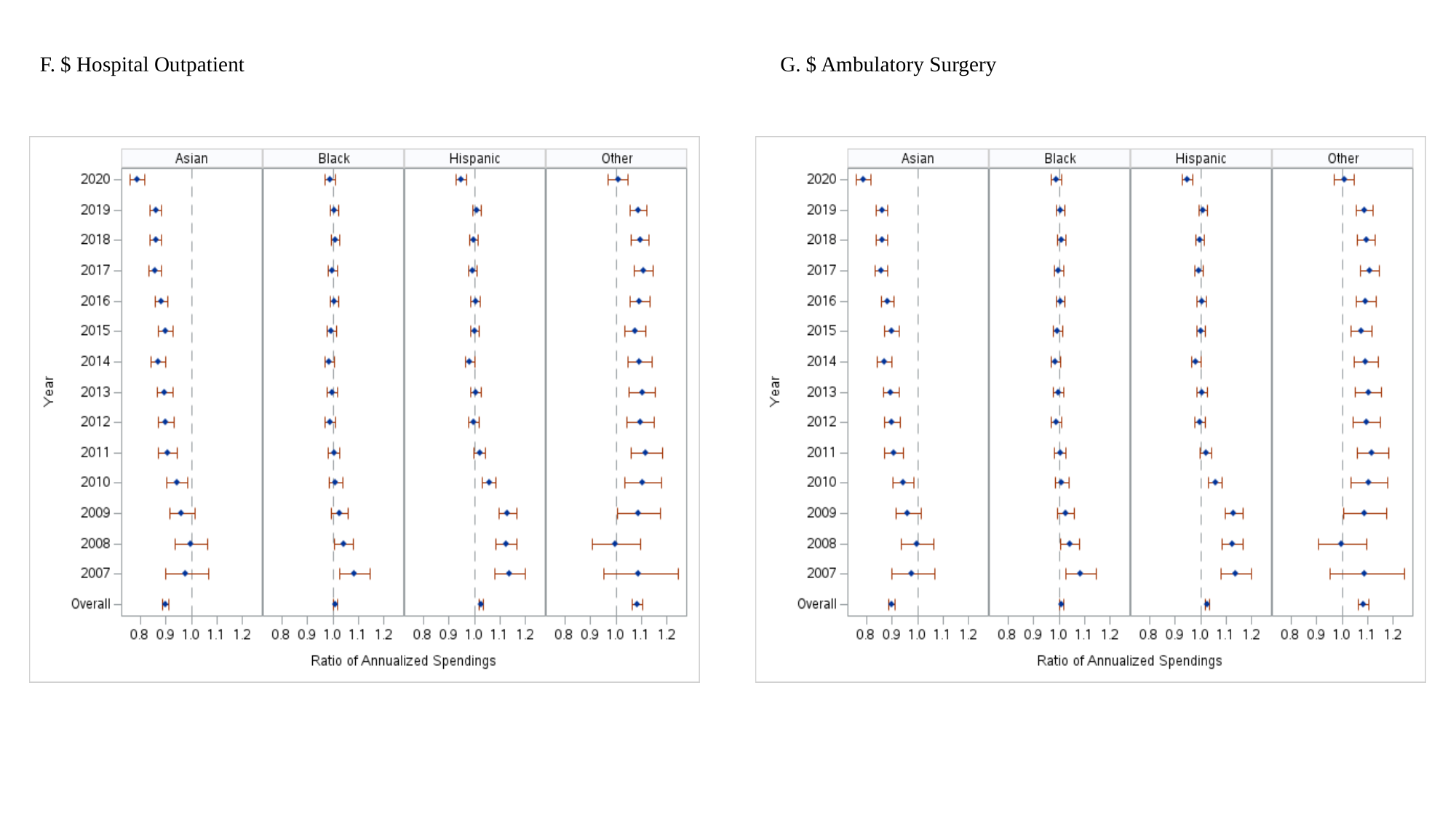

G. $ Ambulatory Surgery
F. $ Hospital Outpatient

## Slide 5
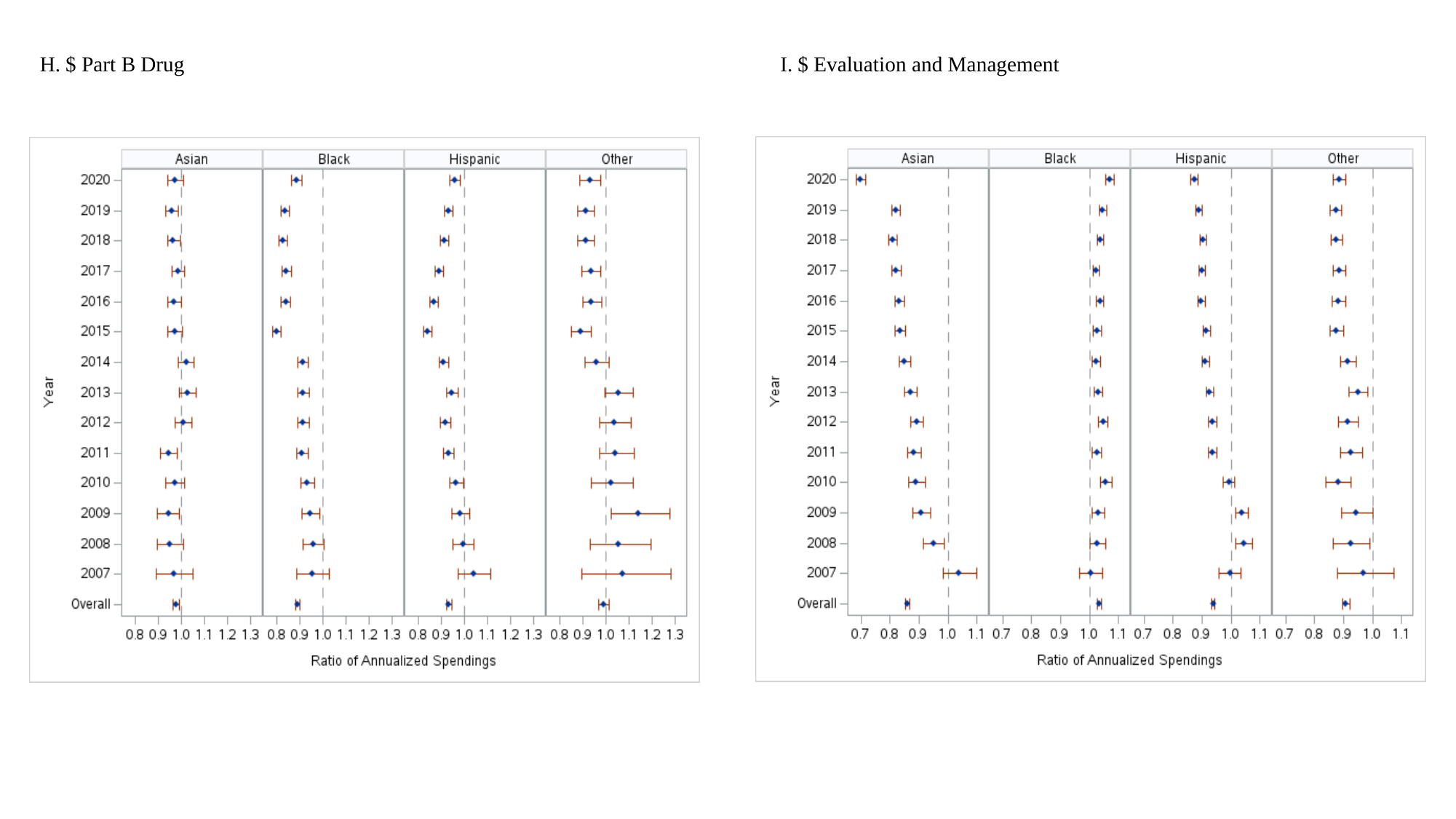

I. $ Evaluation and Management
H. $ Part B Drug

## Slide 6
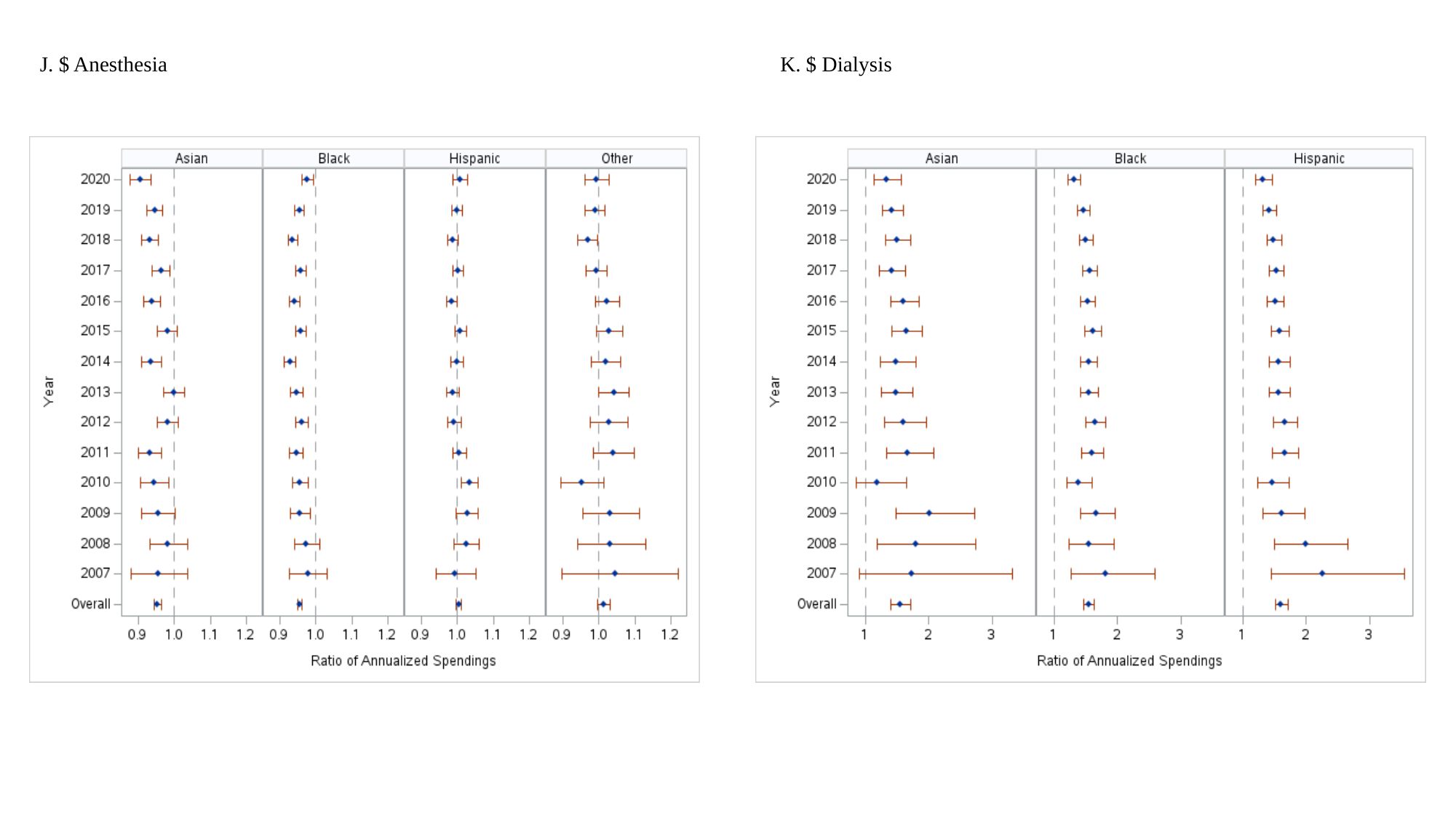

K. $ Dialysis
J. $ Anesthesia

## Slide 7
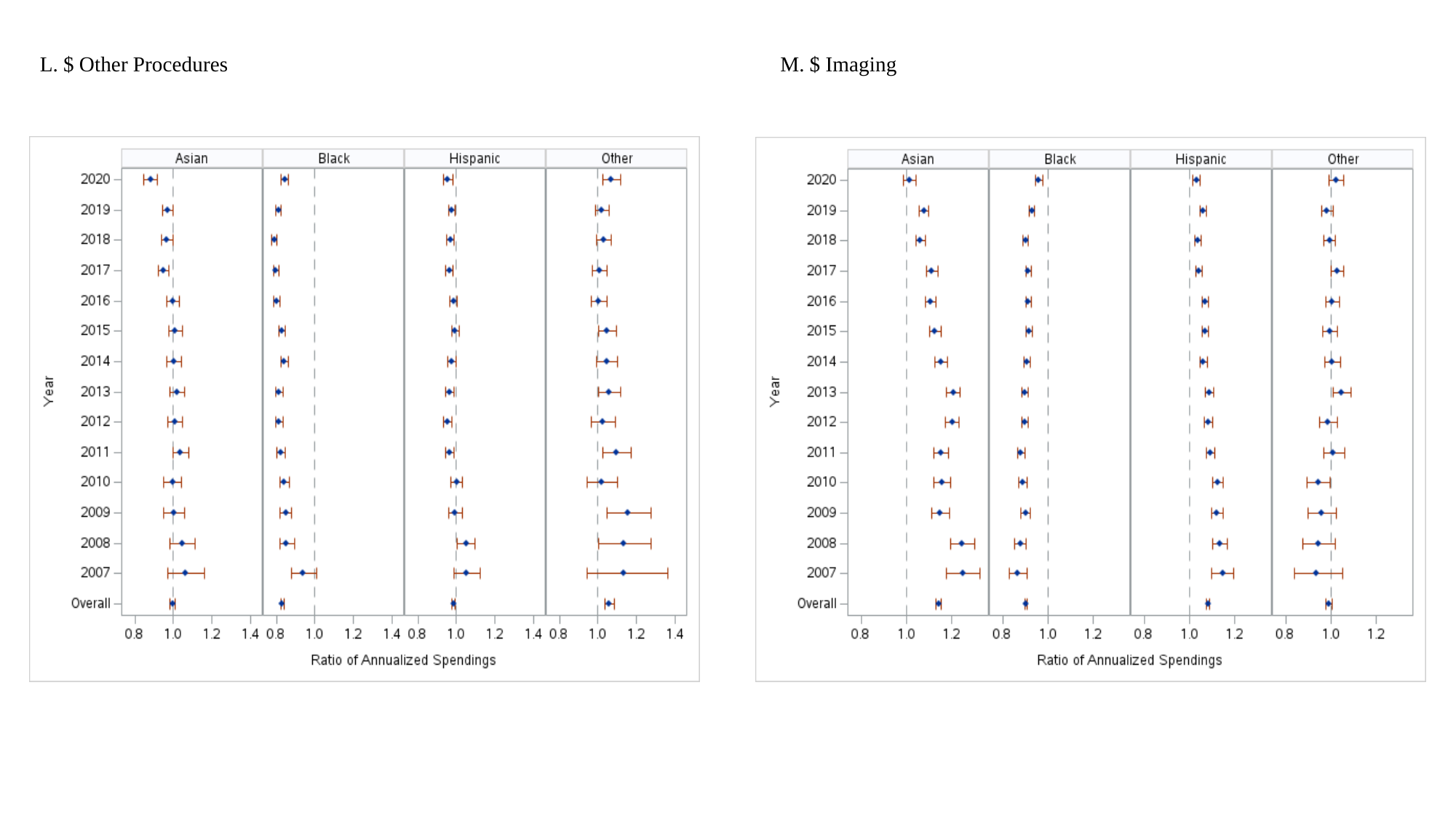

M. $ Imaging
L. $ Other Procedures

## Slide 8
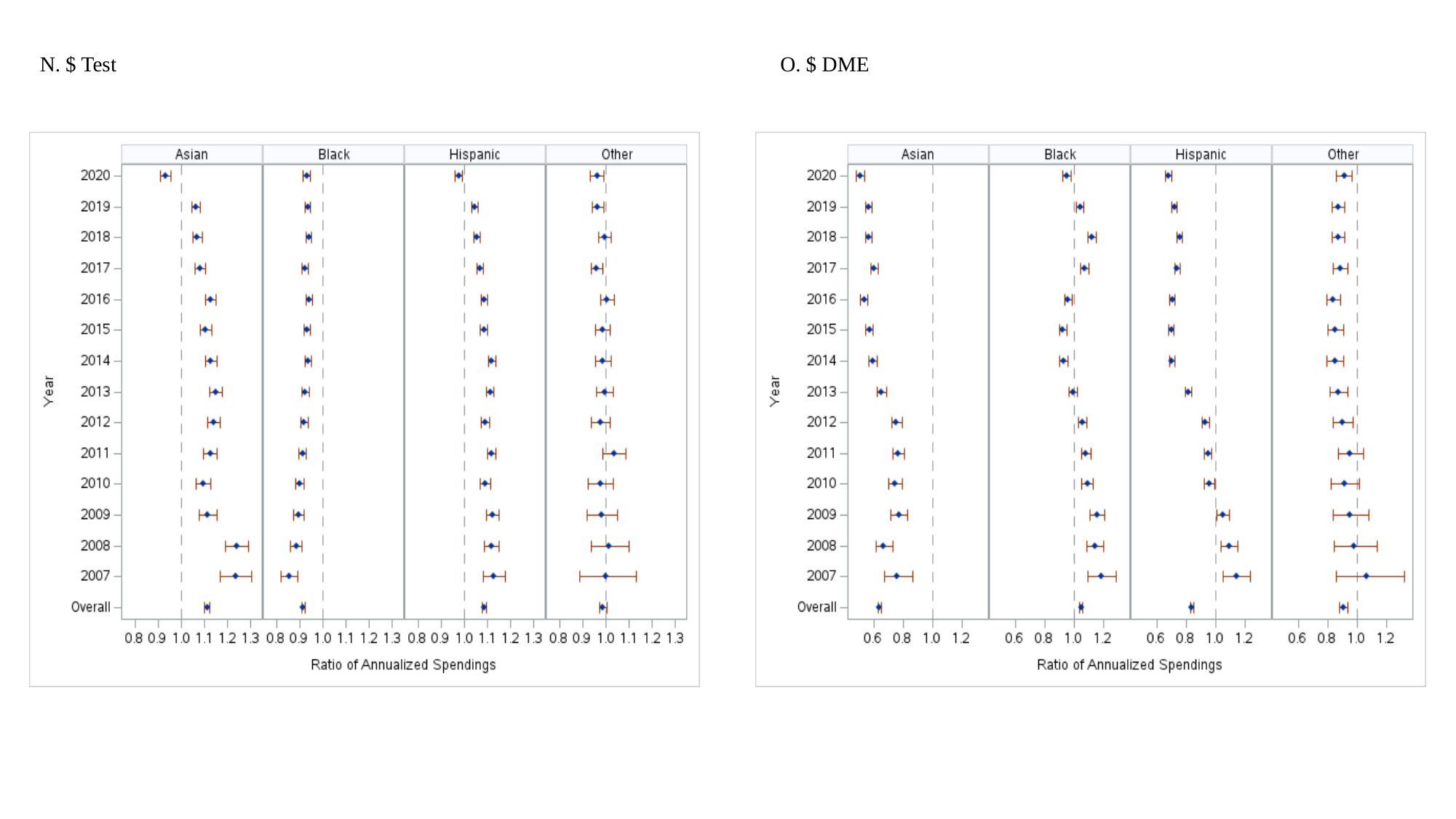

O. $ DME
N. $ Test

## Slide 9
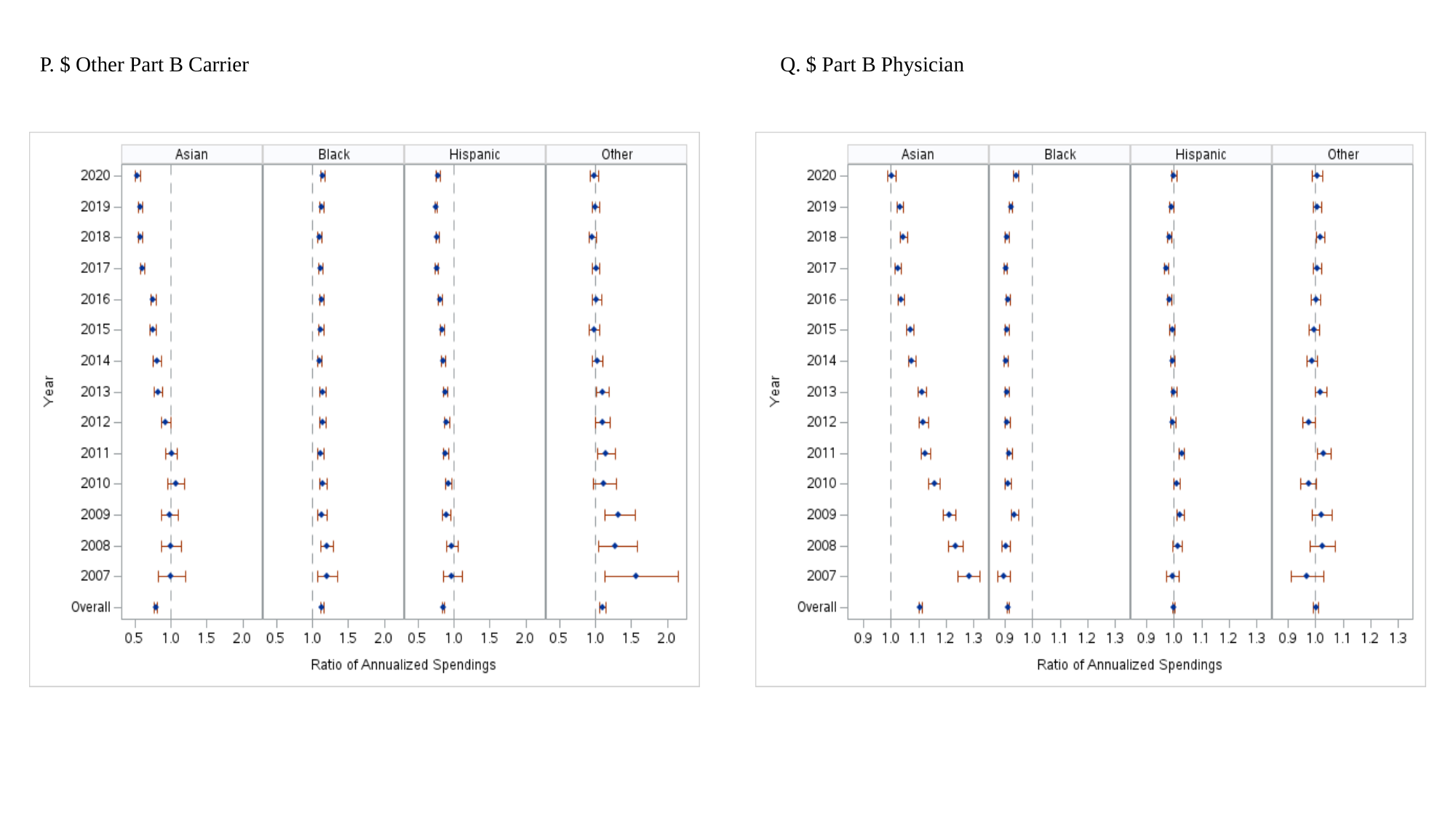

Q. $ Part B Physician
P. $ Other Part B Carrier
