# Supplementary material for: Trends in Racial Disparities in Healthcare Expenditures Among Senior Medicare Fee-for-service Enrollees in 2007–2020
Source: J Racial Ethn Health Disparities. 2023 Nov 13;11(6):3807–17. doi: 10.1007/s40615-023-01832-x (PMC11564202; doi:10.1007/s40615-023-01832-x)
Supplement: Supplementary file 3 — Supplementary file3 (PPTX 288 KB) [file 40615_2023_1832_MOESM3_ESM.pptx]

## Slide 1
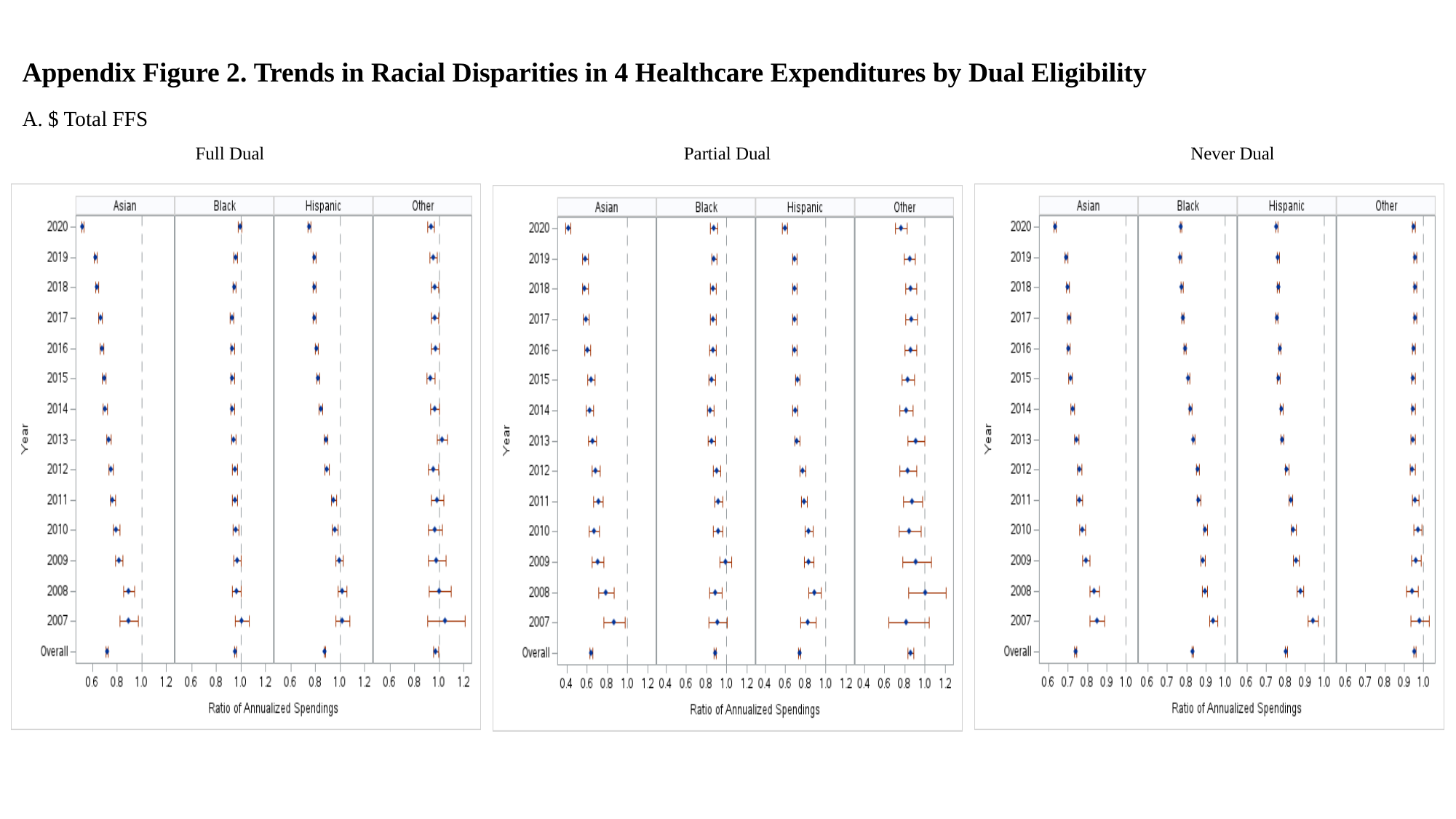

Appendix Figure 2. Trends in Racial Disparities in 4 Healthcare Expenditures by Dual Eligibility
A. $ Total FFS
Partial Dual
Never Dual
Full Dual

## Slide 2
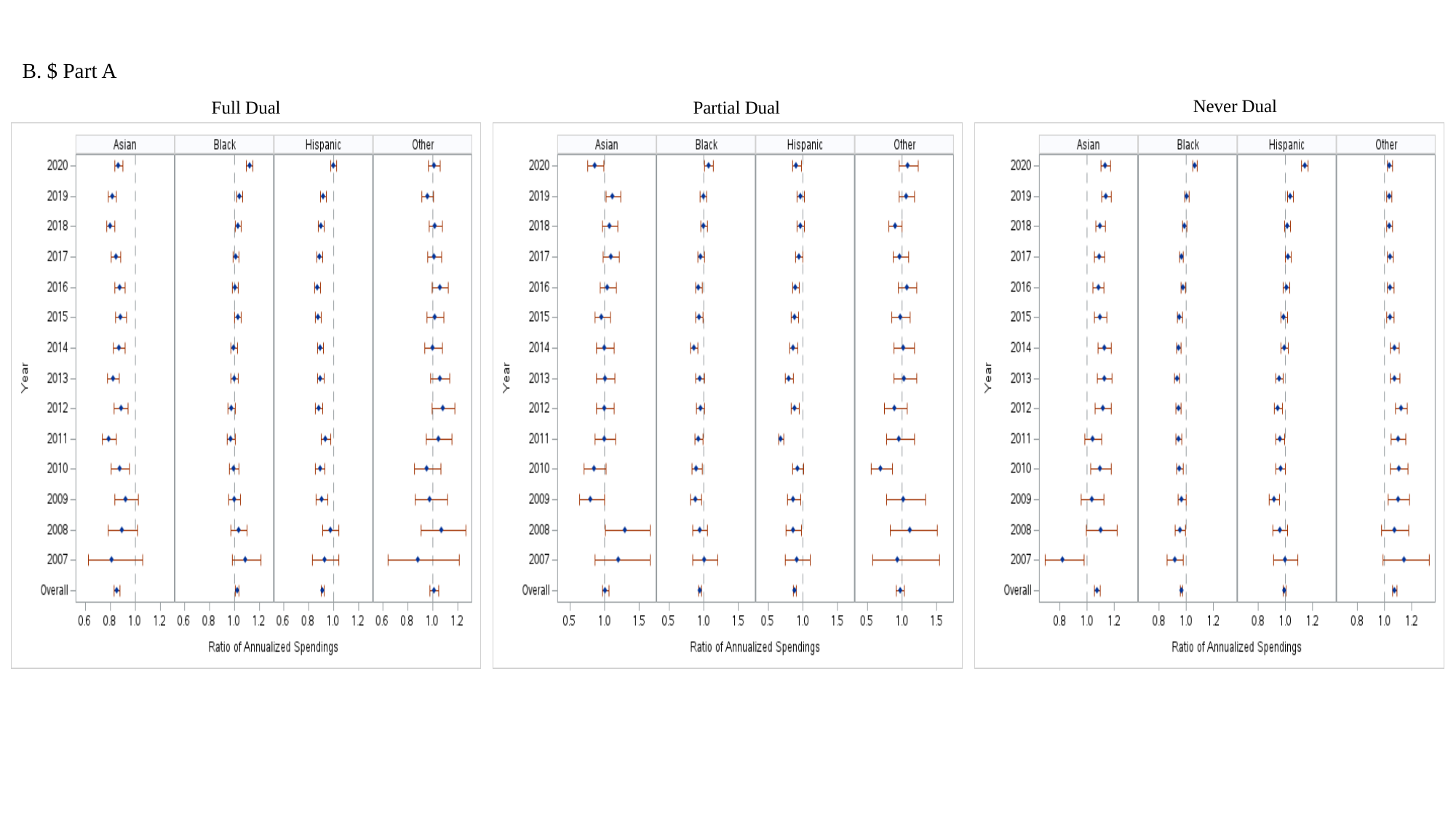

B. $ Part A
Never Dual
Partial Dual
Full Dual

## Slide 3
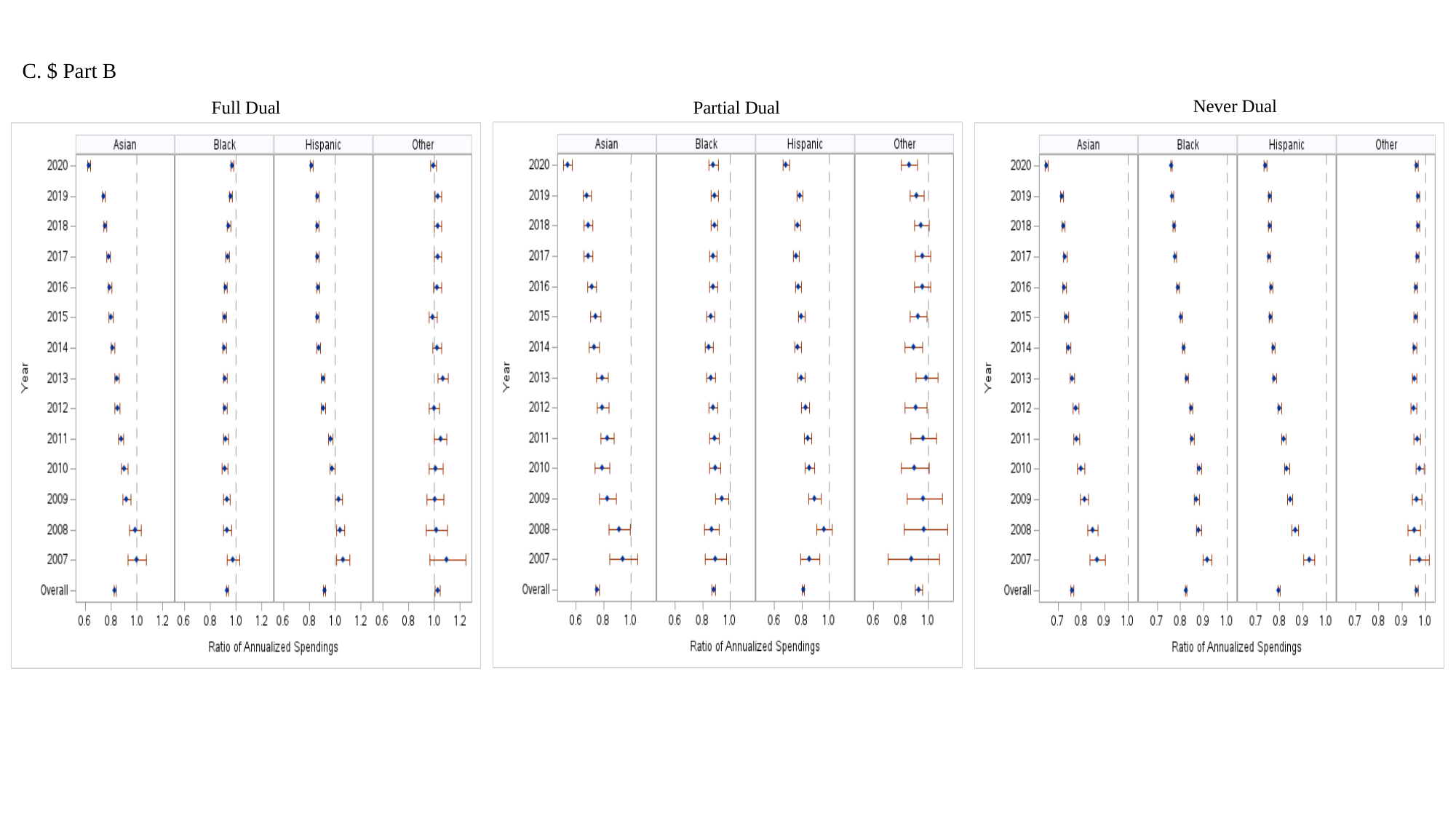

C. $ Part B
Never Dual
Partial Dual
Full Dual

## Slide 4
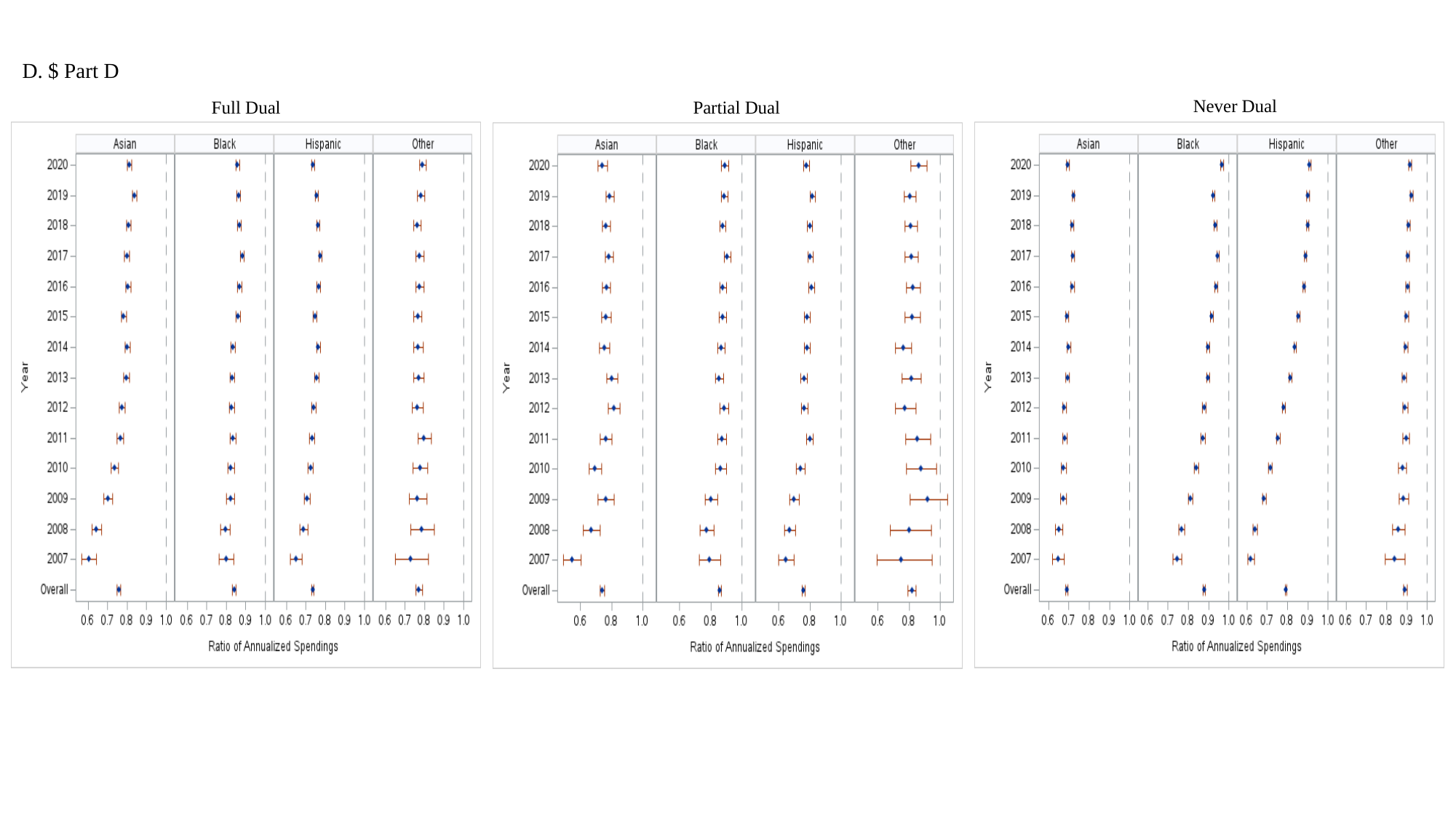

D. $ Part D
Never Dual
Partial Dual
Full Dual
