# Supplementary material for: Trends in Racial Disparities in Healthcare Expenditures Among Senior Medicare Fee-for-service Enrollees in 2007–2020
Source: J Racial Ethn Health Disparities. 2023 Nov 13;11(6):3807–17. doi: 10.1007/s40615-023-01832-x (PMC11564202; doi:10.1007/s40615-023-01832-x)
Supplement: Supplementary file 4 — Supplementary file4 (DOCX 23 KB) [file 40615_2023_1832_MOESM4_ESM.docx]

**Appendix Table 1.** **Size of study sample by year and race**

|  | **Total** | **White** | **Black** | **Hispanic** | **Asian** | **Other** |
| --- | --- | --- | --- | --- | --- | --- |
| **2007** | 424,092 | 343,155 | 32,594 | 30,011 | 11,818 | 6,514 |
| **2008** | 854,666 | 690,503 | 65,426 | 61,379 | 23,842 | 13,516 |
| **2009** | 1,267,482 | 1,018,345 | 97,731 | 93,169 | 36,913 | 21,324 |
| **2010** | 1,674,090 | 1,334,415 | 130,473 | 127,362 | 50,347 | 31,493 |
| **2011** | 2,157,672 | 1,712,363 | 166,324 | 164,511 | 65,740 | 48,734 |
| **2012** | 2,693,682 | 2,124,999 | 206,481 | 204,352 | 83,416 | 74,434 |
| **2013** | 3,195,777 | 2,499,338 | 247,473 | 244,208 | 102,259 | 102,499 |
| **2014** | 3,689,880 | 2,863,526 | 288,964 | 285,472 | 121,923 | 129,995 |
| **2015** | 4,178,081 | 3,219,236 | 331,699 | 327,950 | 142,517 | 156,679 |
| **2016** | 4,681,044 | 3,589,709 | 375,423 | 371,753 | 162,597 | 181,562 |
| **2017** | 5,189,084 | 3,964,859 | 418,622 | 415,052 | 184,991 | 205,560 |
| **2018** | 5,691,101 | 4,331,976 | 462,618 | 462,276 | 207,711 | 226,520 |
| **2019** | 6,199,987 | 4,702,336 | 507,648 | 511,586 | 232,745 | 245,672 |
| **2020** | 6,697,286 | 5,060,234 | 552,696 | 562,455 | 257,267 | 264,634 |

**Notes:** Actual sizes of different expenditure cohorts vary by different qualification requirements such as “enrolled in fee-for-service plan or Part D plan” and “non-zero expenditure”.

**Appendix Table 2. Components of each of 17 total expenditures**

|  |  | **Primary payer** | **Medicare** | **Beneficiary** | **Per-diem** |
| --- | --- | --- | --- | --- | --- |
| **Part A** | **Acute Inpatient** | ✓ | ✓ | ✓ | ✓ |
|  | **Other Inpatient Costs** | ✓ | ✓ | ✓ | ✓ |
|  | **Skilled Nursing Facility** | ✓ | ✓ | ✓ |  |
|  | **Hospice** | ✓ | ✓ |  |  |
|  | **Home Health** | ✓ | ✓ |  |  |
| **Part B** | **Hospital Outpatient** | ✓ | ✓ | ✓ |  |
|  | **Ambulatory Surgery** | ✓ | ✓ | ✓ |  |
|  | **Part B Drug** | ✓ | ✓ | ✓ |  |
|  | **Evaluation and Management** | ✓ | ✓ | ✓ |  |
|  | **Anesthesia** | ✓ | ✓ | ✓ |  |
|  | **Dialysis** | ✓ | ✓ | ✓ |  |
|  | **Other Procedures** | ✓ | ✓ | ✓ |  |
|  | **Imaging** | ✓ | ✓ | ✓ |  |
|  | **Tests** | ✓ | ✓ | ✓ |  |
|  | **Durable Medical Equipment** | ✓ | ✓ | ✓ |  |
|  | **Other Part B Carrier** | ✓ | ✓ | ✓ |  |
|  | **Part B Physician** | ✓ | ✓ | ✓ |  |

**Appendix Table 3. Comparisons of Racial Disparities in Parts A, B, and D expenditure stratified by gender.**

|  | **Female** | **Male** |
| --- | --- | --- |
| **Part A Expenditure** |  |  |
| **Asian vs. White** | 0.939(0.914, 0.964) | 1.019(0.991, 1.048) |
| **Black vs. White** | 0.976(0.965, 0.988) | 0.974(0.961, 0.987) |
| **Hispanic vs. White** | 0.908(0.896, 0.920) | 0.952(0.937, 0.966) |
| **Other vs. White** | 1.004(0.972, 1.038) | 1.029(0.995, 1.065) |
| **Part B Expenditure** |  |  |
| **Asian vs. White** | 0.774(0.766, 0.782) | 0.789(0.779, 0.800) |
| **Black vs. White** | 0.875(0.868, 0.881) | 0.881(0.873, 0.889) |
| **Hispanic vs. White** | 0.852(0.846, 0.858) | 0.825(0.817, 0.832) |
| **Other vs. White** | 0.944(0.929, 0.960) | 0.993(0.972, 1.014) |
| **Part D Expenditure** |  |  |
| **Asian vs. White** | 0.702(0.692, 0.713) | 0.817(0.802, 0.833) |
| **Black vs. White** | 0.865(0.856, 0.874) | 0.814(0.803, 0.825) |
| **Hispanic vs. White** | 0.768(0.760, 0.776) | 0.702(0.693, 0.712) |
| **Other vs. White** | 0.771(0.753, 0.790) | 0.893(0.868, 0.918) |

Note: Data are presented as a ratio of estimated annualized spendings (95% confidence interval).

**Appendix Table 4. Comparisons of Racial Disparities before and after the implementation of Affordable Care Act.**

|  | **Part A** | **Part B** | **Part D** |
| --- | --- | --- | --- |
| **Asian vs. White** | 0.989(0.971, 1.008) | 1.089(1.082, 1.096) | 0.960(0.954, 0.966) |
| **Black vs. White** | 0.985(0.976, 0.993) | 1.019(1.014, 1.024) | 0.963(0.959, 0.967) |
| **Hispanic vs. White** | 0.976(0.967, 0.986) | 1.061(1.056, 1.065) | 0.940(0.937, 0.944) |
| **Other vs. White** | 0.996(0.974, 1.020) | 1.006(0.994, 1.017) | 0.996(0.984, 1.007) |

Notes: We tested statistical significance of difference-in-difference, (disparities in 2014-2020) - (disparities in 2007-2013).
